# Influence of Heat Treatment of Nickel–Titanium Instruments on Cyclic Fatigue Resistance in Simulated Curved Canals

**DOI:** 10.1055/s-0042-1747952

**Published:** 2022-10-04

**Authors:** Gustavo Oliveira Campos, Carlos Eduardo Fontana, Victor Talarico Leal Vieira, Carlos Nelson Elias, Alexandre Sigrist de Martin, Carlos Eduardo da Silveira Bueno

**Affiliations:** 1 Department of Endodontics, School of Dentistry, Universidade Federal de Minas Gerais, Belo Horizonte, Minas Gerais, Brazil; 2 Department of Center for Healthy Sciences, Postgraduate Program in Health Sciences, Pontifícia Universidade Católica, Campinas, São Paulo, Brazil; 3 Department of Odontology, Grande Rio University, Duque de Caxias, Rio de Janeiro, Brasil; 4 Departament of Material Science, Instituto Militar de Engenharia, Rio de Janeiro, Brazil; 5 Departament of Endodontics, School of Dentistry, São Leopoldo Mandic, Campinas, São Paulo, Brazil

**Keywords:** endodontics, kinematics, mechanical stress, nitinol, rotation

## Abstract

**Objective** The aim of this study was to compare the cyclic fatigue of heat-treated and non-treated instruments.

**Materials and Methods** Twenty instruments with and without heat treatment from Bassi Logic were evaluated ( *n* = 10). All instruments were subjected to dynamic cyclic fatigue through continuous rotations inside a stainless-steel tube (1.4-mm diameter, 9-mm curvature with 6-mm radius, and 90° angle) using a custom-made device, which performed 1 axial oscillation every 2 seconds with an amplitude of 3 mm, powered by a torque-controlled motor (Silver Reciproc, VDW, Germany), with speed adjusting to 950 rpm and torque to 4 N, according to manufacturer's guidance. The groups were compared using the Mann–Whitney test.

**Result** The fracture time of the heat-treated instruments (97.20 ± 39.94 second and non-treated instruments (14.30 ± 6.78 seconds had statistical differences [ *p* < 0.05]). Heat-treated instruments were 6.8 times more resistant to fatigue than non-treated instruments ( *p* < 0.05).

**Conclusion** Heat treatment provides increased fatigue resistance of NiTi alloy with the same design.

## Introduction

Endodontic instruments are used to shape the root canal system. Several modifications have been made to the materials over time, such as optimization of alloy composition and instrument geometry, always aiming to enhance performance in clinical practice. 1 In 1988, Walia, Brantley and Gerstein 2 introduced the use of nitinol alloy for the endodontic files' fabrication, showing that the nitinol files were more resistant to fracture and two to three times more flexible than stainless steel files. This alloy is characterized by two crystalline phases. The austenite phase has a body-centered cubic structure and it is characterized by greater stiffness and the martensite phase has an ordered monoclinic structure that provides greater flexibility to the instrument. 1 3 4 5 6

Many improvements have been made to nickel–titanium (NiTi) alloys, including heat treatments. These treatments are able to improve the mechanical properties of the NiTi alloy, producing M-Wire instruments, which are characterized by a decrease in transition temperatures between the microstructural phases, with the austenite phase prevailing in the alloy and CM-Wire instruments, which, at room temperature, contain martensite as the main crystalline structure, providing an effect known as the controlled memory effect, the instrument's ability to return to its original shape upon heating. 3 4 5 6 7

Even after scientific advances in the NiTi alloys microstructure, unexpected instrument fractures may occur as a result of deformation or stress that exceeds the metal elastic limit. Fracture can be caused by torsion, when the tip of the instrument is locked in a canal while the shaft continues to rotate, or by fatigue, when the instrument is subjected to several tension-compression cyclic fatigue at the same point. 8 9 10 11 12 13 14 15

Bassi Logic (Bassi Endo, Belo Horizonte, Brazil) is a rotary file system with files available in tip sizes ranging from #15 to #50 and constant tapers of 0.01, 0.03, 0.04, 0.05, and 0.06. The cross-section depends on the taper of the instrument: the 0.01 taper instruments have four cutting edges; the 0.03, 0.05 and 0.06 taper instruments have a double helix; the 0.04 taper instruments have a triple helix; and the 15.03 and 15.05 instruments have a quadrangular section. All instruments are made of CM-Wire alloy. 16 17

To evaluate the effect of heat treatment on instruments resistance to flexural fatigue, the present study compared the cyclic fatigue resistance of Bassi Logic 25.06 instruments, with and without heat treatment, during repeated tension–compression cycles. The instrument's mechanical properties comparison occurs frequently; however, the instrument design standardization does not occur, which becomes an influence factor in the tests. With the acquisition and donation of similar instruments, it was possible to evaluate the real influence that a heat treatment can cause on the resistance to cyclic fatigue without the influence of design characteristics. The null hypothesis was that there would be no difference in cyclic fatigue resistance among the tested instruments.

## Materials and Methods

The sample size was calculated based on the effect sizes found in the study by Menezes et al. 16 A sample size of 6 units per group was necessary to give 80% power to detect a significant difference between groups, with a type I error of 5% and a type II error of 20%. To account for a rate of 20% of possible losses, a final sample size of 8 units per group was required. Of note, a larger sample size would not affect the power of the study.

Twenty Bassi Logic instruments of a size 25 mm, tip #25, and a 0.06 taper were divided into two groups ( *n* = 10): heat-treated group (HTG) and non-treated group (NTG). All instruments were provided by the company. Non-treated instruments were manufactured exclusively for this research. The instruments were cleaned in an ultrasonic bath using acetone. All instruments were initially inspected for their characteristics, surface finishing, and possible pre-existing deformations under scanning electron microscopy (SEM) (FEG Quanta 250, Eindhoven, Netherlands), with magnifications of 200 × , 250 × , 500 × , 1000 × , and 2000 × . For the surface finish, the instruments were evaluated transversally and the fracture surface observation was transversal.

**Fig. 1:**
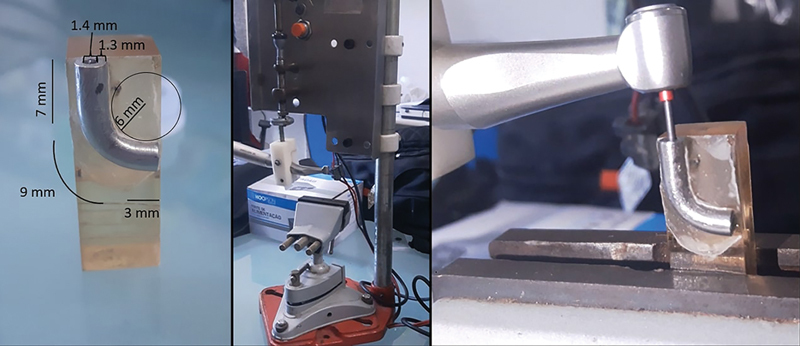
Artificial canal and apparatus used in the test.

The cyclic fatigue test was performed using an artificial canal made out of a stainless steel tube with an inner diameter of 1.4 mm and total length of 19 mm, where the first 7 mm was straight, followed by a 9 mm long curvature with a radius of 6 mm, and ending with a 3 mm straight segment ( Fig. 1 ). An angle of 90° was formed. The canal was attached to a bench vise to avoid unexpected displacement during the test. The apparatus used in the cyclic fatigue test was described previously. 12 18 19 20

Each instrument was activated using a 6:1 reduction contra-angle handpiece Sirona Dental System GmbH, Bensheim, Germany) powered by a torque-controlled motor (Silver Reciproc, VDW, Germany) and pre-set to the “Dr's Choice” mode, adjusting speed to 950 rpm in clockwise rotation and torque to 4 Ncm, following the manufacturer's instruction. The instruments were introduced into the canal until 1 mm of their tip became visible at the end of the tube. Fatigue tests were performed under dynamic conditions, a mechanical device promoted back-and-forth axial movements of the contra-angle handpiece, for a total of 30 vertical oscillations per minute with a 3 mm amplitude of axial movement. 20 The artificial canal lubrication was performed with glycerin (Farmax, Brazil) to reduce the friction between the files and the artificial canal metal. The failure time was recorded in seconds and the confirmation of metal fracture was audibly perceived, and the timer stopped. All instruments were tested at room temperature. The number of cycles until fracture was calculated by multiplying the time (converted to minutes) with the speed used (950 rpm).

After fatigue testing, the fractured instruments were analyzed under SEM to determine the type of fracture, by observing the metal morphological characteristics and the instruments lengths. They were measured with a digital caliper (Digimess, São Paulo, Brazil). To verify the normality of the results, the Shapiro–Wilk test was performed. Due to the fact that the HTG group was not normal, the groups were compared using the Mann–Whitney test. A *p* < 0.05 was considered significant.

## Results

**Fig. 2:**
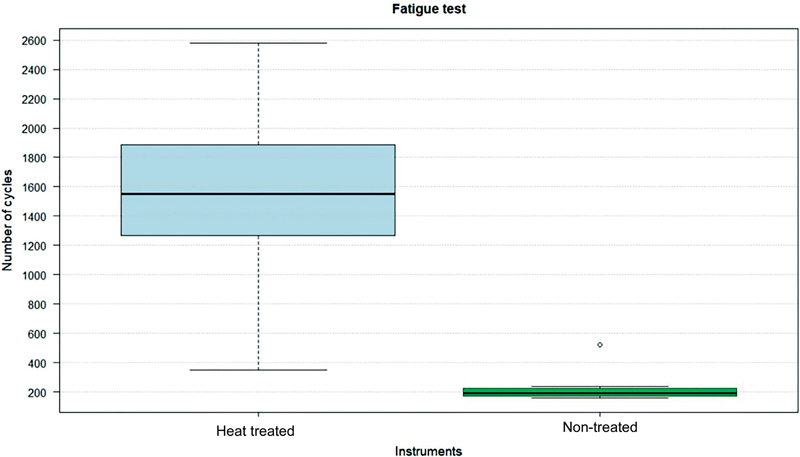
Number of cycles to fracture of heat-treated and non-treated Bassi Logic instruments.

First SEM instruments analysis showed that they were with identical design and were in excellent states of surface finish. Table 1 shows the mean (standard deviation) time to fracture and fragment length for each group. The number of cycles until instruments fracture was calculated by multiplying the time converted in minutes by the engine speed, established by 950 rpm. A comparison of the number of cycles to fracture between the two groups is shown in Fig. 2 . Heat-treated instruments were more resistant to cyclic fatigue failure ( *p* < 0.05), being 6.8 times more resistant to flexural fatigue than non-treated instruments.

**Table 1:** Mean (standard deviation) of time to fracture, number of cycles to fracture (NCF), and fragment length of heat-treated and non–heat-treated Bassi Logic instruments subjected to dynamic cyclic fatigue testing

Instruments	Time (s)	NCF	Fragment length (mm)
Heat-treated	97.20 (39.94) A	1.539 (632) A	4.349 (0.106) A
Non-treated	14.30 (6.78) B	226 (107) B	6.789 (0.160) B

A,B Different superscript letters indicate significant differences ( *p* < 0.05).

**Fig. 3:**
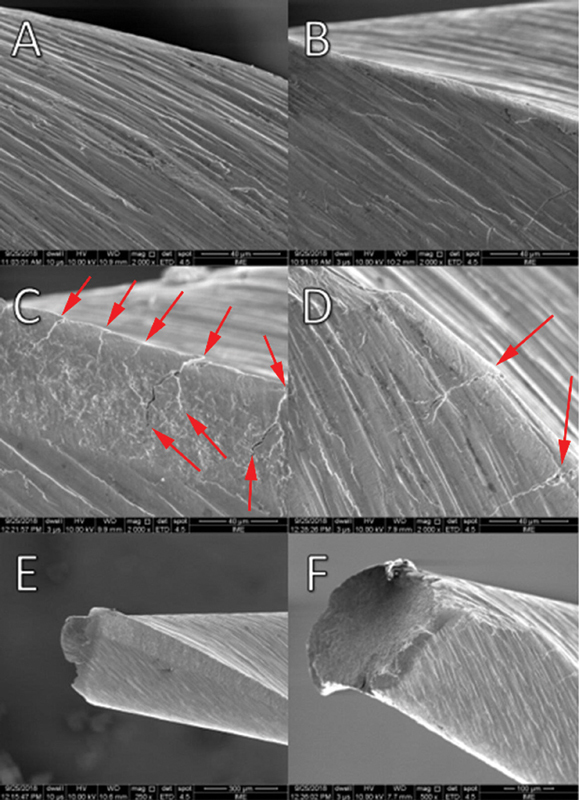
Scanning electron micrographs of ( **A** , **C** , **E** ) non-treated instruments and ( **B** , **D** , **F** ) heat-treated instruments. ( **A** , **B** ) Finishing surface of the tested instruments in 2000 × . ( **C** , **D** ) Presence of several microcracks indicated by arrows in magnification of 2000 × . ( **E** , **F** ) Fracture surfaces showing morphological characteristics of the ductile type in magnification of 200× and 500 × , respectively.

SEM fractured instruments analysis ( Fig. 3 ) revealed that all fractures were caused by the stress and compression cycles of the test, which is characterized by having microvoids that are typical characteristics of ductile fracture, without signs of metal deformation. The microcracks nucleate and propagate in the center region of the stainless-steel tube curvature, this region is where the highest level of stress occurs in the instruments.

## Discussion

The test results were statistically significant, and heat-treated instruments under this study conditions were more resistant to cyclic fatigue failure than non-treated instruments. Therefore, the null hypothesis was rejected.

This is in agreement with previous studies that compared instruments with and without heat treatment. 10 21 22 23 This heat treatment increases the alloy flexibility by decreasing the transition temperatures. 1 By purchasing non-treated instruments directly from the manufacturer, we were able to analyze the direct impact of this treatment on cyclic fatigue resistance in the same instrument, without statistical differences between the groups. The instruments in both groups had exactly the same design and cross-section, differing only in the alloy being heat treated or not.

Multiple factors contribute to increased fatigue resistance, including type of alloy used, treatment received, dynamics of instrument used, instrument design, and the temperature that the tests were performed, in this case, room temperature. 8 9 11 14 24 25 26 27 28 Comparing instruments with strictly identical features at the same room temperature minimizes several biases that may interfere with mechanical performance. 24 25 26 27 28 Fatigue testing can be performed with two types of kinematics, static and dynamic. The choice for dynamic model for this research was because its more closely resembles the daily practice of endodontics, 8 13 20 where instruments are rotated at constant speeds and small vertical amplitudes to minimize the probability of fatigue at only one point along the instrument's long axis, which can speed up fractures. 20

NiTi instruments have great flexibility, allowing the material to recover after small deformations. 2 Heat treatment changes the alloy microstructure and consequently the phase transformation temperatures. The modified transformation temperature is due to the higher fraction of martensite in the heat treated alloy, which makes it more flexible and increases its fatigue life. 29 The heat treatment of the Bassi Logic system transforms a NiTi alloy into a CM-Wire alloy, having a controlled memory and predominating the martensite phase at room temperature, which increases deformation capacity and cyclic fatigue resistance of the alloy. 30 31 Despite the differences in mechanical properties, the clinical results in terms of performance may be similar for the treatment of molars, 32 but the instrument would become safer for the treatment due to the lower level of tension inside the canal, reducing the fracture risk.

This research confirms the results obtained by Keskin et al, 33 who evaluated dynamic cyclic fatigue in Reciproc and Reciproc Blue, instruments with similar design characteristics. They concluded that the instrument with more advanced heat treatment demonstrated greater cyclic fatigue resistance. Another research comparing the cyclic fatigue of Reciproc and Reciproc Blue 21 had the same results, and concluded that the blue treatment optimizes the cyclic fatigue resistance of Reciproc instruments. Elnaghy and Elsaka 10 compared Protaper Universal and Protaper Gold, complemented these studies, demonstrating the increased number of cycles when the NiTi instrument is heat treated and subjected to stress and strain cycles.

The metal fracture occurs at two distinct stages, starting with the formation and multiplication of microcracks followed by crack propagation until final instrument fracture. 34 On the SEM images ( Fig. 3 ), several microcrack sites could be observed along the instrument longitudinal axis resulting from the dynamic movement kinematics.

Rotational speed is a factor that can influence the cyclic fatigue instruments life. 10 18 33 In the current study, speed was standardized at 950 rpm for booth groups and it was not considered a determining factor in the difference between the results for the cycles number to fracture.

Although the instruments were subjected to identical test conditions, there was a difference in fragment length between the two groups ( Table 1 ), which may be explained by the metal malleability. The treated instrument has a higher amount of martensite crystal structure and better ability to incorporate these structures when subjected to high stresses. 13 21 The HTG deforms easier than NTG, supporting the stresses exerted by the metal tube in the mean fracture measurement of the NTG instruments. Comparing the mean fracture length, the difference between the groups remained below the measurement varied by the dynamic movement of 3 mm. The instrument fracture inside a root canal can serve as an obstacle to an adequate root disinfection. Its removal in canals with accentuated curvatures is complicated due to the fact that the fracture occurs in the center of the root curvature, being in a difficult removal position. 35

The SEM images analysis of fracture surfaces showed that fractures were caused by fatigue, without signs of plastic defects in the instrument's axis. Fractures in NTG showed more pronounced cracks in the axis opposite to the site where cracks initiated and propagated. By drawing an association with the difference between fragment lengths, we can assume that this results from the fact that the metal is at higher stress intensity levels at the sharply curved segments with microcracks propagating at a higher speed.

The amount of instruments manufactured for this research was limited only to the fatigue test, complementary tests such as EDS, DSC, DRX, torsion and bending would be interesting to complement the studies regarding the influence of heat treatment on its mechanical performance. For future research, it would also be interesting to perform preparation centering tests on curved teeth.

## Conclusion

Within the limitations of this study, it can be concluded that heat treatment of Bassi Logic instruments improves the resistance to fatigue failure when compared with instruments without heat treatment.
